# Injectable Tranexamic Acid Use in Arthroscopic Rotator Cuff Repair Is Safe and Associated with Reduced Postoperative Opioid Use

**DOI:** 10.3390/jcm15020524

**Published:** 2026-01-08

**Authors:** Ronak J. Mahatme, Shawn A. Moore, Anish Gangavaram, Esha Reddy, Paul McMillan, Brian M. Grawe

**Affiliations:** Department of Orthopaedic Surgery, University of Cincinnati College of Medicine, Cincinnati, OH 45267, USA; mahatmrj@ucmail.uc.edu (R.J.M.); moore6sa@ucmail.uc.edu (S.A.M.); gangavah@mail.uc.edu (A.G.); reddyea@mail.uc.edu (E.R.); mcmillpl@ucmail.uc.edu (P.M.)

**Keywords:** arthroscopic rotator cuff repair, arthroscopy, opioid use, postoperative complications, tranexamic acid

## Abstract

**Background/Objectives:** Tranexamic acid (TXA) is widely used to reduce bleeding in orthopedic surgery, but its safety and impact on outcomes in arthroscopic rotator cuff repair (ARCR) remain unclear. The purpose of this study was to evaluate the safety and effects of injectable TXA on short- and long-term postoperative outcomes and opioid use following ARCR. **Methods:** The TriNetX Research Network, an insurance claims-based database, was utilized to conduct this retrospective, propensity-matched cohort study. Patients aged ≥18 years undergoing ARCR were identified and divided into TXA (*n* = 5855) and non-TXA (*n* = 5855) groups after propensity score matching. Outcomes assessed included 30-day hospital utilization, complications (infection, thromboembolism, hemarthrosis, blood transfusion), one-year revision and shoulder surgery rates, and early, prolonged, and chronic postoperative opioid use. **Results:** No significant differences were observed between groups in 30-day emergency department visits (2.0% vs. 1.8%, *p* = 0.502), readmissions, infections, wound dehiscence, blood transfusions, hemarthrosis, or one-year revision and shoulder surgery rates. TXA use was associated with significantly lower rates of early (24.8% vs. 26.8%, *p* = 0.011), prolonged (9.5% vs. 12.8%, *p* < 0.001), and chronic opioid use (6.6% vs. 9.6%, *p* < 0.001). **Conclusions:** Injectable TXA is safe in ARCR, with no increase in postoperative complications or hospital utilization. Furthermore, TXA use is linked to reduced postoperative opioid consumption, suggesting benefits in pain management and recovery. Prospective studies are warranted to further explore these findings.

## 1. Introduction

Shoulder arthroscopy is a subset of orthopedic procedures that are minimally invasive and can be clinically indicated for numerous injuries including rotator cuff disorders, labral and instability issues, biceps tendon issues, impingement, fractures, and other glenohumeral joint pathologies [1]. As such, common procedures include superior labrum anterior–posterior repair, Bankart repair, capsular plication, biceps tenodesis, subacromial decompression, capsular release, and debridement [2,3]. These procedures are frequently performed in combination during a single operative setting, reflecting the complex and multifactorial nature of shoulder pathology treated arthroscopically.

Specifically, arthroscopic rotator cuff repair (ARCR) is one of the most commonly performed orthopedic sports procedures in the United States, with over 250,000 cases annually [4]. ARCR is often accompanied by management of concomitant long head of the biceps tendon pathology, most commonly through tenotomy or tenodesis, both of which have demonstrated reliable clinical outcomes when performed arthroscopically in conjunction with rotator cuff repair [3]. While ARCR generally yields favorable outcomes, postoperative complications such as infection, wound issues, and venous thromboembolism (VTE) can occur and potentially delay recovery or compromise results [5]. Although the reported incidence of symptomatic VTE after ARCR is low—ranging from 0% to 5.7%, with an overall rate near 0.26% [6]—these events carry significant morbidity if left unrecognized.

Intraoperative bleeding and postoperative hemarthrosis, though typically less severe than in joint arthroplasty, can still complicate ARCR. Poor visualization from bleeding may prolong operative time and impact repair quality, while postoperative swelling and hemarthrosis may contribute to early stiffness, discomfort, and delayed rehabilitation [7,8].

Tranexamic acid (TXA), a synthetic antifibrinolytic agent, works by competitively inhibiting plasminogen activation, thereby stabilizing clot formation and reducing fibrinolysis [9]. In orthopedic procedures such as joint arthroplasty and fracture fixation, TXA has been shown to significantly reduce perioperative blood loss and transfusion requirements without increasing the risk of thromboembolic events [10]. These benefits have sparked growing interest in TXA’s use during ARCR to minimize intra-articular bleeding.

Moreover, TXA may confer additional benefits in ARCR beyond reducing intraoperative bleeding, such as potentially reducing postoperative inflammation [11], pain [12], and early joint swelling [13], which have been implicated in stiffness and delayed rehabilitation following shoulder arthroscopy [14]. Improved intraoperative visualization may also facilitate more efficient tendon mobilization and fixation, potentially reducing operative time and technical complexity [7,15]. These theoretical advantages have led some surgeons to adopt TXA selectively during ARCR, particularly in patients perceived to be at higher risk for bleeding or prolonged operative time.

However, evidence supporting the safety of injectable TXA in shoulder arthroscopy remains limited. Given the low baseline bleeding risk in ARCR, concerns remain about the potential for increased postoperative VTE, infection, or wound complications. Current practice patterns vary, and no formal consensus exists on TXA use in ARCR [7].

The purpose of this study was to evaluate the safety profile of injectable TXA in ARCR by (1) assessing its association with postoperative medical and surgical complications and (2) characterizing complication rates at 30 days, 90 days, and one year. We hypothesized that intraoperative TXA use would not be associated with increased rates of adverse outcomes.

## 2. Materials and Methods

### 2.1. Data Source and Study Design

The data used in this retrospective database cohort study were collected on 18 August 2025 from the TriNetX Research Network, which provided access to electronic medical records (diagnoses, procedures, medications, laboratory values, genomic information) from over 150 million patients from 102 healthcare organizations. TriNetX, LLC is compliant with the Health Insurance Portability and Accountability Act (HIPAA), the US federal law which protects the privacy and security of healthcare data, and any additional data privacy regulations applicable to the contributing HCO. TriNetX is certified to the ISO 27001:2013 standard and maintains an Information Security Management System (ISMS) to ensure the protection of the healthcare data it has access to and to meet the requirements of the HIPAA Security Rule. Any data displayed on the TriNetX Platform in aggregate form, or any patient-level data provided in a data set generated by the TriNetX Platform, only contain de-identified data as per the de-identification standard defined in Section §164.514(a) of the HIPAA Privacy Rule. The process by which the data are de-identified is attested to through a formal determination by a qualified expert as defined in Section §164.514(b)(1) of the HIPAA Privacy Rule. Because this study used only de-identified patient records and did not involve the collection, use, or transmittal of individually identifiable data, this study was exempted from Institutional Review Board approval. Given the retrospective nature of this study using a large administrative database, no priori sample size or power calculation was performed, consistent with STROBE guidelines for observational studies.

### 2.2. Cohort Selection and Inclusion Criteria

Two cohorts were queried for evaluation. The TXA group is defined as patients aged 18 years or older at time of ARCR who received injectable TXA on day of surgery. The non-TXA group is defined as patients aged 18 years or older at time of ARCR who did not receive injectable TXA on day of surgery (Supplementary Table S1).

### 2.3. Exclusion Criteria and Matching Variables

This study excluded patients who had the following prior to surgery: polytrauma, pathological fracture, prior surgical procedures of the shoulder, humerus, and elbow, hemolytic anemia, aplastic anemia, bone marrow failure syndromes, coagulation defects, long term use of anticoagulants, fracture of shoulder/upper arm, malignant neoplasm of bone or articular cartilage, acute kidney failure, chronic kidney disease, pulmonary embolism, and thrombosis. This study was propensity score matched at a 1:1 ratio using the following demographics and comorbidities: age at time of index surgery, sex, race, BMI (>30), tobacco use, osteoporosis, diabetes mellitus, heart failure, and vitamin D deficiency (Supplementary Table S1).

### 2.4. Outcome Measures

The primary outcomes analyzed were hematologic complications, including 30-day postoperative blood transfusions, shoulder hemarthrosis, and 90-day postoperative deep vein thrombosis (DVT) and pulmonary embolism (PE). Secondary outcomes included rates of emergency department (ED) visits, readmissions, infections, wound dehiscence, opioid use, revision procedures, and subsequent shoulder surgeries. Opioid use was determined based on prescription records identified through RxNorm codes (Supplementary Table S2) and stratified into early (1–30 days), prolonged (30–90 days), and chronic (90–180 days) use. Patients with a prior history of DVT, PE, or shoulder hemarthrosis were excluded to prevent confounding by pre-existing conditions. Due to low event rates and the clinical relevance of venous thromboembolism (VTE) following surgery, DVT and PE were combined into a single VTE outcome (Supplementary Table S2).

Findings are presented as risk ratios (RR) with 95% confidence intervals (CI) and associated *p*-values. Hazard ratios (HR) from Cox proportional hazards models are reported when low event counts prevented TriNetX from releasing exact patient numbers. Missing data were absent and adjustments for multiple comparisons were not performed, as the same statistical model was applied across all outcomes. TriNetX rounds event counts less than 11, which may introduce minor imprecision for outcomes affected by this rounding.

## 3. Results

### 3.1. Cohort Identification & Matching

Prior to propensity score matching, 5856 patients aged 18 years and older who underwent arthroscopic rotator cuff repair (ARCR) and received injectable tranexamic acid (TXA) on the day of surgery comprised the TXA group, while 97,765 patients who did not receive TXA comprised the non-TXA group. After matching, each cohort included 5855 patients. Demographic and clinical characteristics of the matched cohorts are summarized in Supplementary Table S3.

### 3.2. 30-Day Outcomes

**Figure 1:**
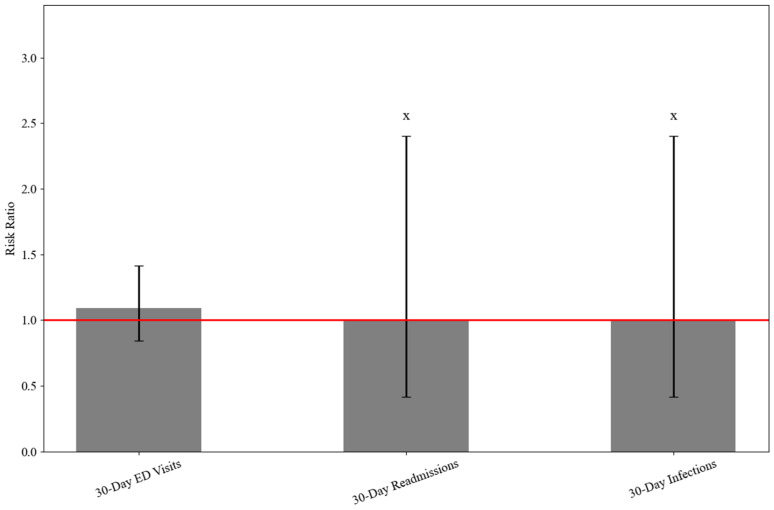
Risk ratios comparing 30-day outcomes between TXA and non-TXA groups during ARCR. X ≤ 10 patients in at least one group due to TriNetX protection of patient information. ED, emergency department.

At 30 days postoperatively, emergency department (ED) visits occurred in 2.0% of TXA patients and 1.8% of non-TXA patients, with no statistically significant difference (RR: 1.093, 95% CI: 0.844–1.415; *p* = 0.502). Readmissions and postprocedural infections were rare (≤10 events per group, 0.2%), showing no significant differences between groups (readmissions RR: 1.000, 95% CI: 0.417–2.401, *p* = 1.000; hazard ratio [HR]: 0.332, 95% CI: 0.067–1.646, *p* = 0.581; infections RR: 1.000, 95% CI: 0.417–2.401, *p* = 1.000; HR: 1.011, 95% CI: 0.293–3.491, *p* = 0.980). Wound dehiscence and blood transfusion each occurred in ≤10 TXA patients (0.2%) and did not occur in the non-TXA group, preventing calculation of RRs for these outcomes. No hemarthrosis cases were reported in either group (Table 1 and Figure 1).

### 3.3. 90-Day Outcomes

**Figure 2:**
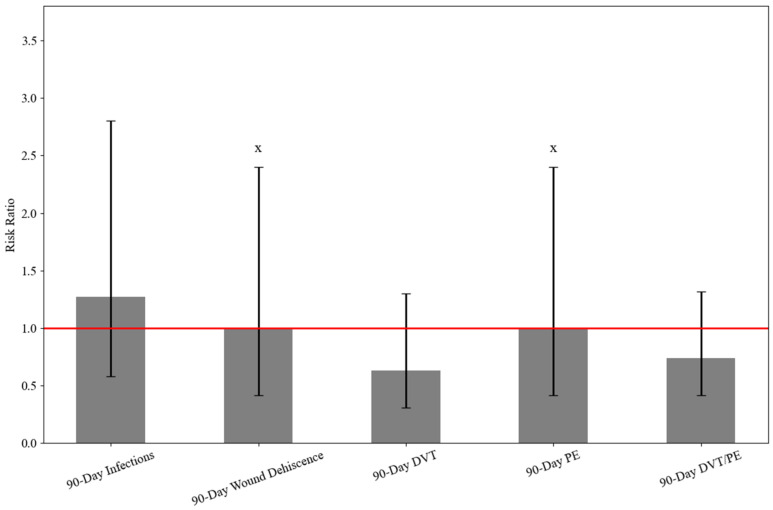
Risk ratios comparing 90-day outcomes between TXA and non-TXA groups during ARCR. X ≤ 10 patients in at least one group due to TriNetX protection of patient information. DVT, deep vein thrombosis; PE, pulmonary embolism.

At 90 days, postprocedural infections occurred in 0.2% of both TXA and non-TXA patients without significant difference (RR: 1.273, 95% CI: 0.578–2.801; *p* = 0.548). Wound dehiscence, deep vein thrombosis (DVT), pulmonary embolism (PE), and combined DVT/PE rates were low (≤0.2–0.5%) and did not differ significantly between groups by RR or HR (all *p* > 0.05) (Table 2 and Figure 2).

### 3.4. One-Year Outcomes

**Figure 3:**
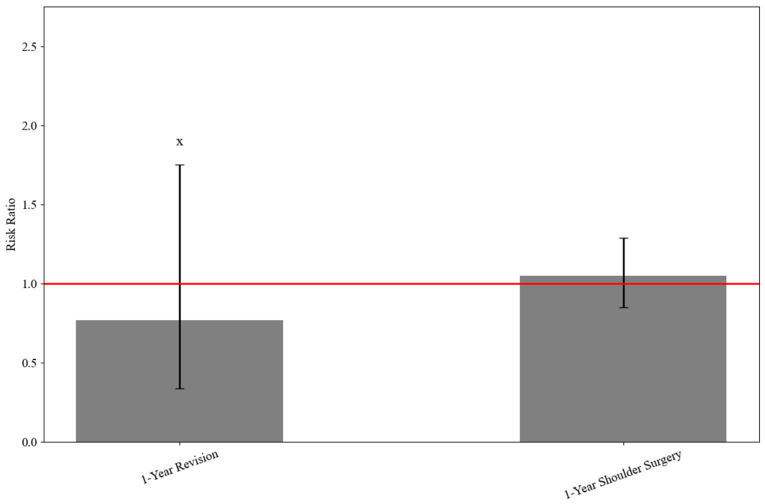
Risk ratios comparing 1-year outcomes between TXA and non-TXA groups during ARCR. X ≤ 10 patients in at least one group due to TriNetX protection of patient information.

One-year revision surgery rates were low and comparable between TXA and non-TXA groups (≤0.2%, RR: 0.769, 95% CI: 0.338–1.753, *p* = 0.531; HR: 0.870, 95% CI: 0.371–2.037, *p* = 0.386). Rates of any shoulder surgery at 1 year were also similar (3.0% vs. 2.9%, RR: 1.048, 95% CI: 0.850–1.291, *p* = 0.661) (Table 3 and Figure 3).

### 3.5. Early, Prolonged, and Chronic Postoperative Opioid Use

**Figure 4:**
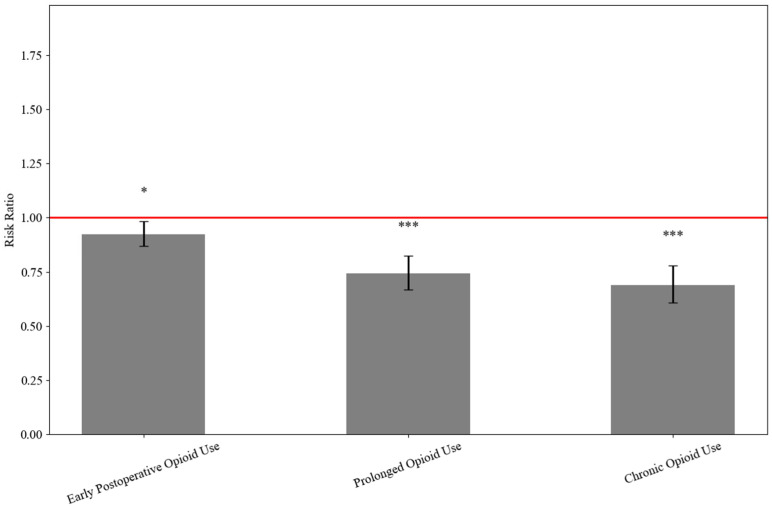
Risk ratios comparing opioid use between TXA and non-TXA groups during ARCR. * < 0.05; ** < 0.01; *** < 0.001.

Compared to non-TXA patients, those receiving TXA had significantly lower rates of early postoperative opioid use (24.8% vs. 26.8%; RR: 0.924, 95% CI: 0.868–0.982; *p* = 0.011) defined as opioid use within 30 days postoperatively, prolonged opioid use (9.5% vs. 12.8%; RR: 0.742, 95% CI: 0.669–0.823; *p* < 0.001) defined as opioid use between 30 and 90 days postoperative, and chronic opioid use (6.6% vs. 9.6%; RR: 0.689, 95% CI: 0.608–0.780; *p* < 0.001) defined as opioid use extending beyond 90 days postoperatively (Table 4 and Figure 4).

## 4. Discussion

This study represents the largest cohort analysis to date examining the safety and impact of intraoperative tranexamic acid (TXA) administration during arthroscopic rotator cuff repair (ARCR). In a propensity-matched population of over 10,000 patients, TXA use was not associated with increased rates of short-term or long-term complications, including thromboembolic events, infections, wound complications, hemarthrosis, or reoperation. Importantly, TXA was associated with significantly reduced early, prolonged, and chronic postoperative opioid use. These findings reinforce the safety profile of TXA in arthroscopic shoulder surgery and suggest a potential role in minimizing postoperative pain and opioid exposure.

As the number of ARCR procedures continues to rise [16], attention has increasingly focused on enhancing surgical efficiency and postoperative recovery [17,18,19,20,21]. TXA, a synthetic antifibrinolytic agent, has become standard in many orthopedic procedures for its ability to reduce perioperative blood loss without increasing the risk of thromboembolic events [22]. Our findings are consistent with prior studies demonstrating the safety of TXA in this regard, as no differences were observed in 90-day rates of deep vein thrombosis, pulmonary embolism, or combined VTE events between TXA and non-TXA groups.

Postoperative wound complications, including infections, dehiscence, and hemarthrosis, were similarly rare and did not differ between groups. These findings likely reflect both the minimally invasive nature of arthroscopic techniques and the low baseline incidence of such complications in ARCR [23]. While prior studies have reported decreased hemarthrosis and transfusion rates in arthroplasty and arthroscopy with TXA [24,25], our study did not identify significant differences, likely due to the already low risk of bleeding in this population.

No differences were observed in 30-day emergency department visits or readmissions between cohorts, further supporting the safety of TXA and suggesting that its use does not contribute to increased short-term healthcare utilization. Similarly, at one-year follow-up, rates of revision surgery and any subsequent shoulder procedures were not significantly different between groups. These findings align with previous literature suggesting TXA does not influence long-term outcomes [17,26].

A key novel finding in our study was the significant reduction in opioid use at multiple time points following surgery in patients receiving TXA. Specifically, TXA use was associated with reduced early (within 30 days), prolonged (30–90 days), and chronic (>90 days) postoperative opioid use. This information is consistent with emerging evidence that TXA may provide short-term improvements in surgical field visualization, reduce intraoperative bleeding, and lessen soft tissue trauma, which may in turn contribute to reduced postoperative pain [7,15,17,27,28,29,30]. Prior studies have shown improved range of motion and pain scores in the early postoperative period following TXA use in ARCR [31], which may explain the reduced need for opioids during critical phases of recovery. Early reductions in pain and inflammation may have a meaningful impact on functional recovery and opioid stewardship.

It is also important to acknowledge that the observed reduction in postoperative opioid use among patients receiving TXA may reflect factors beyond its direct pharmacologic effects. TXA use may be more common among surgeons or institutions that employ enhanced recovery pathways, standardized multimodal analgesia protocols, or more proactive opioid-sparing pain management strategies. In this context, TXA administration may serve as a surrogate for broader perioperative practice patterns that emphasize improved pain control, early mobilization, and judicious opioid prescribing. Nonetheless, regardless of the underlying mechanism, the association between TXA use and reduced opioid exposure remains clinically meaningful, highlights an important area for future prospective investigation, and suggests that TXA could be considered as part of multimodal strategies to optimize postoperative pain management in ARCR.

Animal models have also suggested that TXA may reduce postoperative adhesions and improve early range of motion [32], offering a potential mechanistic explanation for our observed reduction in opioid requirements. Given the clinical and public health implications of postoperative opioid use, particularly in the context of the opioid epidemic, our findings support further investigation into TXA as a potential adjunct to multimodal pain management in ARCR.

This study has limitations inherent to retrospective database analyses. Despite rigorous propensity matching, residual confounding may persist especially due to surgeon-specific practices and broader institutional or system-level factors that may have influenced pain management, recovery pathways, or opioid prescribing patterns. Additionally, the TriNetX platform is limited by the accuracy of administrative coding (ICD-10, CPT, and RxNorm) and lacks granular clinical detail, including TXA timepoint (preoperative, intraoperative, or postoperative), administration (intra-articular vs. intravenous), dosage, tear characteristics, surgical technique, or specific rehabilitation protocols. In addition, opioid prescription does not equate to consumption and opioid indication (surgical vs. non-surgical pain) cannot be verified. Furthermore, small event counts (<11) are rounded per TriNetX privacy guidelines, limiting precision for some outcomes. Moreover, patient-reported outcomes and functional recovery following ARCR in our patient population are unknown. Finally, as a database study, TriNetX data may overrepresent larger academic or integrated health care systems, thereby limiting generalizability. Nonetheless, this study provides the most comprehensive and generalizable data to date on TXA use in ARCR, with a large, matched cohort and robust outcome assessment across multiple time points.

Future prospective studies are warranted to better characterize granular clinical variables related to TXA administration, including timing, route of delivery (systemic versus intra-articular), dosage, and intended indication (e.g., hemostasis or visualization), which may clarify its optimal clinical applicability and further elucidate the mechanisms underlying reduced postoperative opioid use following ARCR.

## 5. Conclusions

This large-scale, propensity-matched analysis demonstrates that injectable tranexamic acid (TXA) use during arthroscopic rotator cuff repair (ARCR) is safe and not associated with increased risks of short-term postoperative complications, including hospital utilization, thromboembolic events, infections, hemarthrosis, or blood transfusions. Furthermore, TXA use was linked to significantly reduced early, prolonged, and chronic postoperative opioid consumption, suggesting potential benefits in pain management and recovery. These findings support the safe incorporation of TXA in ARCR and highlight the need for prospective studies to further elucidate its role in improving intraoperative visualization and perioperative recovery.

## Figures and Tables

**Table 1:** Thirty-day outcomes in TXA and non-TXA patients during ARCR. Hazard Ratios are provided when they could be calculated, risk ratios are not significant, and either group’s event count is less than 11. * Event counts are less than 11 and rounded due to TriNetX limitations in reporting small event count data to prevent patient deindividualization. ^ *p*-values may not accurately represent significance as calculations are done with rounded patient populations. TXA, tranexamic acid; RR, risk ratio; CI, confidence interval; HR, hazard ratio; ED, emergency department.

Variable	TXA Events (Risk %)	Non-TXA Events (Risk %)	Absolute Risk Difference (%)	Total Patients Per Cohort	RR (95% CI)	*p*	HR (95% CI)	*p*
30-day ED Visits	118 (2.0)	108 (1.8)	0.2	5855	1.093 (0.844–1.415)	0.502		
30-Day Readmissions	≤10 * (0.2)	≤10 * (0.2)	0	5855	1.000 (0.417–2.401)	1.000 ^	0.332 (0.067–1.646)	0.581
30-Day Postprocedural Infections	≤10 * (0.2)	≤10 * (0.2)	0	5855	1.000 (0.417–2.401)	1.000 ^	1.011 (0.293–3.491)	0.980
30-day Wound Dehiscence	≤10 * (0.2)	0 (0.0)	0.2	5855	-	-		
30-Day Blood Transfusions	≤10 * (0.2)	0 (0.0)	0.2	5855	-	-		
30-Day Hemarthrosis	0 (0.0)	0 (0.0)	0	5853/5855	-	-		

**Table 2:** Ninety-day outcomes in TXA and non-TXA patients during ARCR. Hazard Ratios are provided when able to be calculated, risk ratios are not significant, and either group’s event count is less than 11. * Event counts are less than 11 and rounded due to TrinetX limitations in reporting small event count data to prevent patient deindividualization. ^ *p*-values may not accurately represent significance as calculations are done with rounded patient populations. TXA, tranexamic acid; RR, risk ratio; CI, confidence interval; HR, hazard ratio; DVT, deep vein thrombosis; PE, pulmonary embolism.

Variable	TXA Events (Risk %)	Non-TXA Events (Risk %)	Absolute Risk Difference (%)	Total Patients Per Cohort	RR (95% CI)	*p*	HR (95% CI)	*p*
90-day Postprocedural Infections	14 (0.2)	11 (0.2)	0	5855	1.273 (0.578–2.801)	0.548		
90-day wound dehiscence	≤10 * (0.2)	≤10 * (0.2)	0	5855	1.000 (0.417–2.401)	1.000 ^	2.077 (0.188–22.909)	0.100
90-day DVT	12 (0.2)	19 (0.3)	0.1	5855	0.632 (0.307–1.300)	0.208		
90-day PE	≤10 * (0.2)	≤10 * (0.2)	0	5855	1.000 (0.417–2.401)	1.000 ^	1.128 (0.435–2.923)	0.348
90-day DVT/PE	20 (0.3)	27 (0.5)	0.2	5855	0.741 (0.416–1.319)	0.306		

**Table 3:** One-year outcomes in TXA and non-TXA patients during ARCR. Hazard Ratios are provided when they could be calculated, risk ratios are not significant, and either group’s event count is less than 11. * Event counts are less than 11 and rounded due to TriNetX limitations in reporting small event count data to prevent patient deindividualization. ^ *p*-values may not accurately represent significance as calculations are done with rounded patient populations. TXA, tranexamic acid; RR, risk ratio; HR, hazard ratio; CI, confidence interval.

Variable	TXA Events (Risk %)	Non-TXA Events (Risk %)	Absolute Risk Difference (%)	Total Patients Per Cohort	RR (95% CI)	*p*	HR (95% CI)	*p*
1-Year Revision	≤10 * (0.2)	13 (0.2)	0	5855	0.769 (0.338–1.753)	0.531 ^	0.870 (0.371–2.037)	0.386
1-Year Shoulder Surgery	175 (3.0)	167 (2.9)	0.1	5855	1.048 (0.850–1.291)	0.661		

**Table 4:** Early, prolonged, and chronic postoperative opioid use in TXA and non-TXA patients during ARCR. TXA, tranexamic acid; RR, risk ratio; CI, confidence interval.

Variable	TXA Events (Risk %)	Non-TXA Events (Risk %)	Absolute Risk Difference (%)	Total Patients Per Cohort	RR (95% CI)	*p*
Early Postoperative Opioid Use	1450 (24.8)	1570 (26.8)	2.0	5855	0.924 (0.868–0.982)	**0.011**
Prolonged Postoperative Opioid Use	554 (9.5)	747 (12.8)	3.3	5855	0.742 (0.669–0.823)	**<0.001**
Chronic Postoperative Opioid Use	387 (6.6)	562 (9.6)	3.0	5855	0.689 (0.608–0.780)	**<0.001**

## Data Availability

The data used in this retrospective cohort study were collected from the TriNetX Research Network.

## Supplementary Materials

The following supporting information can be downloaded at: https://www.mdpi.com/article/10.3390/jcm15020524/s1, Table S1: CPT, ICD-10-CM, RxNorm codes utilized for inclusion, exclusion, and propensity score matching.; Table S2: CPT, ICD-10-PCS, and ICD-10-CM Codes for Outcome Variables; Table S3: Propensity Score Matching of TXA and non-TXA during ARCR.
