# Supplementary material for: Injectable Tranexamic Acid Use in Arthroscopic Rotator Cuff Repair Is Safe and Associated with Reduced Postoperative Opioid Use
Source: J Clin Med. 2026 Jan 8;15(2):524. doi: 10.3390/jcm15020524 (PMC12842260; doi:10.3390/jcm15020524)
Supplement: Supplementary file 1 [file jcm-15-00524-s001.zip › Supplementary Table S1.pdf]

Supplementary Table S1: CPT, ICD-10-CM, RxNorm codes utilized for inclusion, exclusion, and propensity score matching.

Item	CPT Code	ICD-10-CM Code	RxNorm
**Inclusion**			
Arthroscopic Shoulder Surgery with Rotator Cuff Repair (> 18 years old)	29827		
Tranexamic Acid – Injectable Product			10691
**Exclusions from all Cohorts if Prior to Surgery**			
Unspecified Multiple Injuries		T07	
Osteoporosis with Current Pathological Fracture		M80	
Surgical Procedures on the Shoulder	1004147		
Surgical Procedures on the Humerus and Elbow	1004279		
Arthroscopic Shoulder Surgery with Rotator Cuff Repair	1005614		
Hemolytic Anemias		D55-D59	
Aplastic and Other Anemias and Other Bone Marrow Failure Syndromes		D60-D64	
Coagulation Defects, Purpura, and Other Hemorrhagic Conditions		D65-D69	
Other Disorders of Blood and Blood-Forming Organs		D70-D77	
Long Term (Current) Use of Anticoagulants		Z79.01	
Fracture of Shoulder and Upper Arm		S42	
Malignant Neoplasms of Bone and Articular Cartilage		C40-C41	
Acute Kidney Failure and Chronic Kidney Disease		N17-N19	
Pulmonary Embolism		I26	
Phlebitis and Thrombophlebitis		I80	
Other Venous Embolism and Thrombosis		I82	
Cerebrovascular Diseases		I60-I69	
Embolism and Thrombosis of Arteries of the Lower Extremities		I74.3	
Thrombosis due to Internal Orthopedic Prosthetic Devices, Implants and Grafts		T84.86	
Embolism due to Internal Orthopedic Prosthetic Devices, Implants, and Grafts		T84.81	
**Propensity score matching inputs**			
Osteoporosis without current pathological fracture		M81	
Vitamin D deficiency		E55	

BMI > 30		Z68.3, Z68.4	
Tobacco use		Z72.0	
Diabetes Mellitus		E08-E13	
Heart Failure		I50	
