# Supplementary material for: Injectable Tranexamic Acid Use in Arthroscopic Rotator Cuff Repair Is Safe and Associated with Reduced Postoperative Opioid Use
Source: J Clin Med. 2026 Jan 8;15(2):524. doi: 10.3390/jcm15020524 (PMC12842260; doi:10.3390/jcm15020524)
Supplement: Supplementary file 1 [file jcm-15-00524-s001.zip › Supplementary Table S2.pdf]

Supplementary Table S2: CPT, ICD-10-PCS, and ICD-10-CM Codes for Outcome Variables

**ED Visits**		
**Outcome definition**		
Procedure	UMLS:CPT:99281	Emergency department visit for the evaluation and management of a patient that may not require the presence of a physician or other qualified health care professional
Procedure	UMLS:CPT:99282	Emergency department visit for the evaluation and management of a patient, which requires a medically appropriate history and/or examination and straightforward medical decision making
Procedure	UMLS:CPT:99283	Emergency department visit for the evaluation and management of a patient, which requires a medically appropriate history and/or examination and low level of medical decision making
Procedure	UMLS:CPT:99284	Emergency department visit for the evaluation and management of a patient, which requires a medically appropriate history and/or examination and moderate level of medical decision making
Procedure	UMLS:CPT:99285	Emergency department visit for the evaluation and management of a patient, which requires a medically appropriate history and/or examination and high level of medical decision making
**Settings for the performed analyses**		
Risk analysis		including patients with outcome prior to the time window
Kaplan - Meier survival analysis		including patients with outcome prior to the time window
**Readmissions**		
**Outcome definition**		
Procedure	UMLS:CPT:1013699	Inpatient or Observation Consultations
**Settings for the performed analyses**		
Kaplan - Meier survival analysis		including patients with outcome prior to the time window
Risk analysis		including patients with outcome prior to the time window
**Postprocedural Infection**		
**Outcome definition**		
Diagnosis	UMLS:ICD10CM:T81.4	Infection following a procedure
**Settings for the performed analyses**		
Risk analysis		including patients with outcome prior to the time window
Kaplan - Meier survival analysis		including patients with outcome prior to the time window
**Wound Dehiscence**		
**Outcome definition**		
Diagnosis	UMLS:ICD10CM:T81.30	Disruption of wound, unspecified
Diagnosis	UMLS:ICD10CM:T81.31	Disruption of external operation (surgical) wound, not elsewhere classified
Diagnosis	UMLS:ICD10CM:T81.32	Disruption of internal operation (surgical) wound, not elsewhere classified
**Settings for the performed analyses**		
Kaplan - Meier survival analysis		including patients with outcome prior to the time window
Risk analysis		including patients with outcome prior to the time window
**Opioid Use**		
**Outcome definition**		
Medication	NLM:RXNORM:5489	hydrocodone

Medication	NLM:RXNORM:6813	methadone
Medication	NLM:RXNORM:7814	oxymorphone
Medication	NLM:RXNORM:7052	morphine
Medication	NLM:RXNORM:3423	hydromorphone
Medication	NLM:RXNORM:7804	oxycodone
Medication	NLM:RXNORM:6754	meperidine
Medication	NLM:RXNORM:480	alfentanil
Medication	NLM:RXNORM:2670	codeine
Medication	NLM:RXNORM:4337	fentanyl
Medication	NLM:RXNORM:4166	ethylmorphine
Medication	NLM:RXNORM:787390	tapentadol
Medication	NLM:RXNORM:56795	sufentanil
Medication	NLM:RXNORM:7242	naloxone
Medication	NLM:RXNORM:10689	tramadol
Medication	NLM:RXNORM:1551777	naloxegol
Medication	NLM:RXNORM:29899	methylnaltrexone
Medication	NLM:RXNORM:6378	levorphanol
Medication	NLM:RXNORM:7243	naltrexone
Medication	NLM:RXNORM:7238	nalbuphine
Medication	NLM:RXNORM:1819	buprenorphine
Medication	NLM:RXNORM:3500	diphenoxylate
Medication	NLM:RXNORM:6468	loperamide
Medication	NLM:RXNORM:73032	remifentanil
**Settings for the performed analyses**		
Kaplan - Meier survival analysis		including patients with outcome prior to the time window
Risk analysis		including patients with outcome prior to the time window
**Postoperative Hemarthrosis**		
**Outcome definition**		
Diagnosis	UMLS:ICD10CM:M25.01	Hemarthrosis, shoulder
**Settings for the performed analyses**		
Kaplan - Meier survival analysis		excluding patients with outcome prior to the time window
Risk analysis		excluding patients with outcome prior to the time window

**Blood Transfusions**		
**Outcome definition**		
Procedure	UMLS:CPT:36430	Transfusion, blood or blood components
Procedure	UMLS:HCPCS:P9010	Blood (whole), for transfusion, per unit
Procedure	UMLS:HCPCS:P9011	Blood, split unit
Procedure	UMLS:HCPCS:P9012	Cryoprecipitate, each unit

Procedure	UMLS:HCPCS:P9016	Red blood cells, leukocytes reduced, each unit
Procedure	UMLS:HCPCS:P9017	Fresh frozen plasma (single donor), frozen within 8 hours of collection, each unit
Procedure	UMLS:HCPCS:P9019	Platelets, each unit
Procedure	UMLS:HCPCS:P9020	Platelet rich plasma, each unit
Procedure	UMLS:HCPCS:P9021	Red blood cells, each unit
Procedure	UMLS:HCPCS:P9022	Red blood cells, washed, each unit
Procedure	UMLS:HCPCS:P9023	Plasma, pooled multiple donor, solvent/detergent treated, frozen, each unit
Procedure	UMLS:HCPCS:P9026	Cryoprecipitated fibrinogen complex, pathogen reduced, each unit
Procedure	UMLS:HCPCS:P9031	Platelets, leukocytes reduced, each unit
Procedure	UMLS:HCPCS:P9032	Platelets, irradiated, each unit
Procedure	UMLS:HCPCS:P9033	Platelets, leukocytes reduced, irradiated, each unit
Procedure	UMLS:HCPCS:P9034	Platelets, pheresis, each unit
Procedure	UMLS:HCPCS:P9035	Platelets, pheresis, leukocytes reduced, each unit
Procedure	UMLS:HCPCS:P9036	Platelets, pheresis, irradiated, each unit
Procedure	UMLS:HCPCS:P9037	Platelets, pheresis, leukocytes reduced, irradiated, each unit
Procedure	UMLS:HCPCS:P9038	Red blood cells, irradiated, each unit
Procedure	UMLS:HCPCS:P9039	Red blood cells, deglycerolized, each unit
**Settings for the performed analyses**		
Kaplan - Meier survival analysis		including patients with outcome prior to the time window
Risk analysis		including patients with outcome prior to the time window

**Wound Dehiscence**		
**Outcome definition**		
Diagnosis	UMLS:ICD10CM:T81.30	Disruption of wound, unspecified
Diagnosis	UMLS:ICD10CM:T81.31	Disruption of external operation (surgical) wound, not elsewhere classified
Diagnosis	UMLS:ICD10CM:T81.32	Disruption of internal operation (surgical) wound, not elsewhere classified
**Settings for the performed analyses**		
Risk analysis		including patients with outcome prior to the time window
Kaplan - Meier survival analysis		including patients with outcome prior to the time window
**Postprocedural Infection**		
**Outcome definition**		
Diagnosis	UMLS:ICD10CM:T81.4	Infection following a procedure
**Settings for the performed analyses**		
Risk analysis		including patients with outcome prior to the time window
Kaplan - Meier survival analysis		including patients with outcome prior to the time window
**DVT**		
**Outcome definition**		
Diagnosis	UMLS:ICD10CM:I82	Other venous embolism and thrombosis
Diagnosis	UMLS:ICD10CM:T84.86	Thrombosis due to internal orthopedic prosthetic devices, implants and grafts

Diagnosis	UMLS:ICD10CM:T84.81	Embolism due to internal orthopedic prosthetic devices, implants and grafts
Diagnosis	UMLS:ICD10CM:I80.1	Phlebitis and thrombophlebitis of femoral vein
Diagnosis	UMLS:ICD10CM:I80.2	Phlebitis and thrombophlebitis of other and unspecified deep vessels of lower extremities
Diagnosis	UMLS:ICD9CM:451.83	Phlebitis and thrombophlebitis of deep veins of upper extremities
**Settings for the performed analyses**		
Kaplan - Meier survival analysis		Excluding patients with outcome prior to the time window
Risk analysis		Excluding patients with outcome prior to the time window
**PE**		
**Outcome definition**		
Diagnosis	UMLS:ICD10CM:I26	Pulmonary embolism
**Settings for the performed analyses**		
Kaplan - Meier survival analysis		excluding patients with outcome prior to the time window
Risk analysis		excluding patients with outcome prior to the time window
**DVT/PE**		
**Outcome definition**		
Diagnosis	UMLS:ICD10CM:I26	Pulmonary embolism
Diagnosis	UMLS:ICD10CM:I82	Other venous embolism and thrombosis
Diagnosis	UMLS:ICD10CM:T84.86	Thrombosis due to internal orthopedic prosthetic devices, implants and grafts
Diagnosis	UMLS:ICD10CM:T84.81	Embolism due to internal orthopedic prosthetic devices, implants and grafts
Diagnosis	UMLS:ICD10CM:I80	Phlebitis and thrombophlebitis
**Settings for the performed analyses**		
Kaplan - Meier survival analysis		excluding patients with outcome prior to the time window
Risk analysis		excluding patients with outcome prior to the time window

**Revision**				
	**Outcome definition**			
		Procedure	UMLS:CPT:23420	Reconstruction of complete shoulder (rotator) cuff avulsion, chronic (includes acromioplasty)
		Procedure	UMLS:CPT:29827	Arthroscopy, shoulder, surgical; with rotator cuff repair
		Procedure	UMLS:CPT:23412	Repair of ruptured musculotendinous cuff (eg, rotator cuff) open; chronic
	**Settings for the performed analyses**			
		Risk analysis		including patients with outcome prior to the time window
		Kaplan - Meier survival analysis		including patients with outcome prior to the time window
**Surgical Procedures on the Shoulder**				

**Outcome definition**			
	Procedure	UMLS:CPT:1004147	Surgical Procedures on the Shoulder
	Procedure	1005614	Arthroscopy, shoulder, surgical
**Settings for the performed analyses**			
	Risk analysis		including patients with outcome prior to the time window
	Kaplan - Meier survival analysis		including patients with outcome prior to the time window
