# Supplementary material for: Injectable Tranexamic Acid Use in Arthroscopic Rotator Cuff Repair Is Safe and Associated with Reduced Postoperative Opioid Use
Source: J Clin Med. 2026 Jan 8;15(2):524. doi: 10.3390/jcm15020524 (PMC12842260; doi:10.3390/jcm15020524)
Supplement: Supplementary file 1 [file jcm-15-00524-s001.zip › Supplementary Table S3.pdf]

Supplementary Table S3: Propensity Score Matching of TXA and non-TXA during ARCR

**Cohort 1 and cohort 2 patient count before and after propensity score matching**		
Cohort	Patient count before matching	Patient count after matching
1 – TXA ARCR	5,856	5,855
2 – non-TXA ARCR	97,765	5,855

Cohort 1 (N = 5,856) and cohort 2 (N = 97,765) characteristics before propensity score matching

Demographics

Cohort			Mean ± SD	Patients	% of Cohort	P-Value	Std diff.
1<br>2	AI	Age at Index	58.8 +/- 10.0<br>57.6 +/- 10.2	5,856<br>97,193	100%<br>100%	<0.001	0.125
1<br>2	2106-3	White		4,565<br>70,581	78.0%<br>72.6%	<0.001	0.124
1<br>2	1002-5	American Indian or Alaska Native		27<br>453	0.5%<br>0.5%	0.956	0.001
1<br>2	F	Female		2,446<br>38,528	41.8%<br>39.6%	0.001	0.043
1<br>2	2076-8	Native Hawaiian or Other Pacific Islander		25<br>420	0.4%<br>0.4%	0.953	0.001
1<br>2	2054-5	Black or African American		495<br>9,505	8.5%<br>9.8%	0.001	0.046
1<br>2	M	Male		3,319<br>54,758	56.7%<br>56.3%	0.613	0.007
1<br>2	2131-1	Other Race		170<br>3,741	2.9%<br>3.8%	<0.001	0.052
1<br>2	2028-9	Asian		218<br>2,489	3.7%<br>2.6%	<0.001	0.067

Diagnosis

Cohort			Mean ± SD	Patients	% of Cohort	P-Value	Std diff.
1<br>2	Z68.3	Body mass index [BMI] 30-39, adult		628<br>7,006	10.7%<br>7.2%	<0.001	0.123
1<br>2	Z68.4	Body mass index [BMI] 40 or greater, adult		235<br>2,496	4.0%<br>2.6%	<0.001	0.081
1<br>2	E08-E13	Diabetes mellitus		670<br>10,694	11.4%<br>11.0%	0.298	0.014
1<br>2	Z72.0	Tobacco use		169<br>2,112	2.9%<br>2.2%	<0.001	0.045
1<br>2	M81	Osteoporosis without current pathological fracture		153<br>2,205	2.6%<br>2.3%	0.087	0.022
1<br>2	E55	Vitamin D deficiency		585<br>7,480	10.0%<br>7.7%	<0.001	0.081
1<br>2	N18	Chronic kidney disease (CKD)		0<br>0	0%<br>0%	--	--
1<br>2	I50	Heart failure		55<br>812	0.9%<br>0.8%	0.399	0.011

Cohort 1 (N = 5,855) and cohort 2 (N = 5,855) characteristics after propensity score matching

Demographics

Cohort			Mean ± SD	Patients	% of Cohort	P-Value	Std diff.
1<br>2	AI	Age at Index	58.8 +/- 10.0<br>59.0 +/- 9.9	5,855<br>5,855	100%<br>100%	0.488	0.013
1<br>2	2106-3	White		4,564<br>4,591	78.0%<br>78.4%	0.546	0.011
1<br>2	1002-5	American Indian or Alaska Native		27<br>24	0.5%<br>0.4%	0.674	0.008
1<br>2	F	Female		2,445<br>2,456	41.8%<br>41.9%	0.837	0.004
1<br>2	2076-8	Native Hawaiian or Other Pacific Islander		25<br>24	0.4%<br>0.4%	0.886	0.003
1<br>2	2054-5	Black or African American		495<br>488	8.5%<br>8.3%	0.816	0.004
1<br>2	M	Male		3,319<br>3,304	56.7%<br>56.4%	0.780	0.005
1<br>2	2131-1	Other Race		170<br>168	2.9%<br>2.9%	0.912	0.002
1<br>2	2028-9	Asian		218<br>206	3.7%<br>3.5%	0.553	0.011

Diagnosis

Cohort			Mean ± SD	Patients	% of Cohort	P-Value	Std diff.
1<br>2	Z68.3	Body mass index [BMI] 30-39, adult		627<br>626	10.7%<br>10.7%	0.976	0.001
1<br>2	Z68.4	Body mass index [BMI] 40 or greater, adult		234<br>215	4.0%<br>3.7%	0.361	0.017
1<br>2	E08-E13	Diabetes mellitus		670<br>623	11.4%<br>10.6%	0.166	0.026
1<br>2	Z72.0	Tobacco use		169<br>170	2.9%<br>2.9%	0.956	0.001
1<br>2	M81	Osteoporosis without current pathological fracture		153<br>151	2.6%<br>2.6%	0.907	0.002
1<br>2	E55	Vitamin D deficiency		584<br>573	10.0%<br>9.8%	0.733	0.006
1<br>2	N18	Chronic kidney disease (CKD)		0<br>0	0%<br>0%	--	--
1<br>2	I50	Heart failure		55<br>51	0.9%<br>0.9%	0.696	0.007
